# Enhancing nutritional status, growth, and fruit quality of dried figs using organic fertilizers in rain-fed orchards: A case study in Estahban, Iran

**DOI:** 10.1371/journal.pone.0300615

**Published:** 2024-04-03

**Authors:** Moslem Jafari, Ali Akbar Ghasemi-Soloklui, Mojtaba Kordrostami

**Affiliations:** 1 Fig Research Station, Fars Agricultural and Natural Resources Research and Education Center, Agricultural Research, Education and Extension Organization (AREEO), Estahban, Iran; 2 Nuclear Agriculture Research School, Nuclear Science and Technology Research Institute (NSTRI), Karaj, Iran; Bahauddin Zakariya University, PAKISTAN

## Abstract

The majority of Iranian fig production is exported, making it one of the world’s most well-known healthy crops. Therefore, the main objective of the current experiment was to investigate the effects of various types of organic fertilizers, such as animal manure (cow and sheep), bird manure (partridge, turkey, quail, and chicken), and vermicompost, on the nutritional status of trees, vegetative and reproductive tree characteristics, fruit yield, and fruit quality traits in dried fig cultivar (“Sabz”). According to the findings, applying organic fertilizers, particularly turkey and quail, significantly improves vegetative and reproductive characteristics. However, other manures such as sheep, chicken, and vermicompost had a similar effect on the growth parameters of fig trees. Additionally, the findings indicated that except for potassium, use of all organic fertilizers had an impact on macro and microelements such as phosphorus, nitrogen, and sodium amount in fig tree leaves. Also, based on fruit color analysis in dried figs, the use of all organic fertilizers improved fruit color. Moreover, the analyses fruit biochemical showed that the use of some organic fertilizers improved that TSS and polyphenol compounds such as coumarin, vanillin, hesperidin gallic acid and trans frolic acid. In general, the results indicated that the addition of organic fertilizers, especially turkey manure, led to increased vegetative productivity and improvement in the fruit quality of the rain-fed fig orchard.

## Introduction

Growing figs (*Ficus carica* L.) is an important agricultural and economic practice that provides revenue, employment, and nutrition for people all over the world [1]. Dry fig is one of the most important fruit crops grown in arid and semi-arid regions of Iran, and Iran is one of the largest dried fig production centers in the world [2]. Additionally, the rain-fed orchards in Estahban represent a typical fig-growing region in Iran, and responsible for the majority of fig production (17,000 tonnes of figs) in Iran [3]. Estahban has an average annual rainfall of 350 mm and is situated in the province of Fars to the southeast of Iran. The primary commercially cultivated dried fig variety in Iran is known as “Sabz”, and it is grown in rain-fed fig orchards.

The majority of Iranian fig production is exported, making it one of the most well-known healthy crops in the world. Moreover, consumers prefer products that have not been chemically treated. Additionally, the demand for safe fruit has been growing recently and is expected to continue in the future, especially when it is grown using agricultural practices that maintain the product’s quality and guarantee food safety. Due to low input utilization in both traditional and natural management approaches, yield and quality of fig fruit in Estahban orchards is usually decreasing. This problem can be more easily recognized in inorganic production, where there is greater knowledge transfer from contracting companies. One of the finest management practices for improving soil fertility and avoiding dependency on mineral fertilizer in organic fruit production orchards is the use of organic amendments [4]. Organic matter plays a key role in influencing quality by improving the source of macro and microelements in the soil, helping to raise water holding, boosting the carbon (C)/nitrogen (N) ratio in the soil, improving the physical characteristics of the soil, reducing the toxicity of heavy metals, and extending microbial biodiversity [5]. Thus, one of the main techniques for increasing soil fertility as an addition to or replacement for synthetic fertilizers is the use of organic amendments. Additionally, selecting an organic material type with high-quality requirements capable of satisfying the demands of plants and soil is the key challenge for the safe and sustainable usage of organic compounds.

Animal manure, such as sheep and cattle manure, has been frequently utilized for soil fertilization by fig growers over several decades due to the low costs involved in its production, delivery, and processing. Moreover, with its extensive availability and nutritional intake of trace micro and macro elements, it is an appealing choice for improving fertilization on nutritionally deficient soils [1, 2]. Vermicompost is another important component of utilizing organic fertilizers, as they may improve food quality. It is a finely fragmented developed product that resembles peat and has great porosity, aeration, drainage, and water-holding capacity, as well as microbial activity. Vermicompost is rich in soil microorganisms, especially fungi, bacteria, and actinomycetes, and contains macro elements, including nitrates, phosphates, exchangeable calcium, and potassium, as well as plant growth regulators and other materials that influence plant growth, such as humates produced by microorganisms [6]. Various researchers in different countries have used animal manure, particularly from cattle, for fig trees in orchards. Cattle manure fertilization is recommended in fig tree plantations because it has a positive effect on the soil’s physical characteristics, the supply of nutrients, and the colony of nematophagous microorganisms [7, 8]. For instance, Tofanelli, de Jesus [1] in Brazil found that organic fertilization of fig trees with 5 L of cattle manure per tree led to better results in all physiological parameters when compared to the plants that were not treated. Additionally, fig trees fertilized with 2.5 L of cow dung compared to plants treated with 5 L of cattle manure or 1.25 L of bird manure produced figs with a higher maturation index. In Malaysia, Azmi, Tajudin [9] reported that 10% of chicken manure increased the fig trees’ survivability to 100%, and this treatment also produced significantly more branches and leaves than other treatments. The results indicated that adding chicken manure to the soil increased its quality and encouraged fig trees to grow in tropical environments. In Turkey, Mordoğan, Hakerlerler [10] discovered that the measured doses of cow and sheep manure mixes had no noticeable impact on the concentrations of the macronutrients (N, P, K, Ca, and Mg) in the fig leaf lamina. While numerous studies explore organic fig production, the majority focus on irrigated orchards. Limited resources address fig tree nutrition in rain-fed conditions, particularly regarding animal and bird manure utilization. Additionally, no data exist comparing animal and bird manure to other organic fertilizers like vermicompost concerning growth traits, fruit yield, and dried fig cultivar quality. Therefore, the main objective of the current experiment was to investigate the effects of various types of organic fertilization, such as animal manure (cow and sheep), bird manure (partridge, turkey, quail, and chicken), and vermicompost, on the nutritional status of trees, vegetative and reproductive tree characteristics, fruit yield, and fruit quality traits in the important commercial dried fig cultivar “Sabz” with sandy, low-organic matter soils in Estahban, Iran.

## Material and methods

### 2.1. Location and plant material

The study was conducted at the Estahban Fig Research Station in Estahban (Fars province, Iran) from 2019 and 2020. Metrological data for two years presented in Table 1. The location’s coordinates are 29° 07’ N latitude and 54° 04’ E longitude, with an elevation of 1749 m above sea level. Soil samples were taken in October from two depths (0–30 and 30–60 cm), revealing that the experimental soil is slightly acidic, non-saline (EC = 1.23), loamy in texture, moderately alkaline in reaction (pH of 7.53), and low in humus content. The main physical and chemical soil properties of the experimental orchard are presented in Table 2. The selected cultivar for the study was “Sabz” as it is the main commercial and common variety cultivated in Iran. The fig orchard consisted of 35-year-old trees planted at 10x10 meters spacing and trained with multiple trunks (4–8 trunks per tree) in rain-fed conditions. The trees were pollinated to achieve the best caprifig fruit set during the caprification season.

**Table 1:** Climatic parameters of meteorological stations in Estahbann (including 2019 and 2020 years) utilized in the current study based on a ‘‘Red, Yellow and Green color-based scaling system.

	Rainfall (mm)	Humidity (%)		Temperature (°C)		
		Max	Min	Average	Max	Min
	2019					
January	10.2	76.6	29.0	9.1	16.8	1.4
February	37.4	76	31	7.5	13.7	1.3
March	122.5	74.49	30.80	9.94	16.19	3.69
April	84.4	82.2	33.6	15.1	21.99	8.22
May	7.4	65.29	19.22	20.39	29.01	11.78
June	0	38.58	9.96	26.38	35.81	17.51
July	6.6	45	11	29.3	38.2	20.4
August	0	34	10	27.7	35.9	19.4
September	0.3	46	12	25.7	34.8	16.6
October	0.4	60.1	20.8	19.4	27.9	11.0
November	11.8	69	28	10.2	17.7	2.2
December	93.8	85.48	37.64	9.21	17.45	0.96
	**2020**					
January	143.1	87.9	42.5	5.5	11.2	-0.2
February	14.6	70.5	22.7	9.89	17.2	2.56
March	106	71.2	28.1	11.01	17.7	4.27
April	59.21	77.9	32.8	15.99	22.6	9.36
May	1.6	62	16	21.1	29.8	12.4
June	0	43	12	26.3	35.9	16.7
July	5.1	49	15	28.5	36.3	20.7
August	0.7	46	16	29.3	37.2	21.4
September	0	39	12	23	31.2	14.8
October	0.0	45.0	14.0	15.6	24.8	6.5
November	22.7	74	33	12.5	20.3	4.8
December	42	89	36	7.9	14.8	1

**Table 2:** Physical and chemical properties of rain fed fig orchard soil before fertilization (soil samples from depths of 30–70 cm).

Particle Size distribution (%)			Texture class	Ec (dS/m)	pH	Available elements							
Sand	Silt	Clay				Organic Carbon (%)	N (%)	P (mg/kg)	K (mg/kg)	Fe^2+^ (mg/kg)	Mn^2+^ (mg/kg)	Cu^2+^ (mg/kg)	Zn^2+^ (mg/kg)
28	42	30	Loam	1.23	7.53	0.2	0.02	17.1	330.95	2.8	7.8	0.78	3.92

### 2.2. Experimental design and treatments

The experiment followed a randomized block design (RCBD) with eight treatments and three replications (one tree per replication). Three “Sabz” fig trees with similar growth vigor were selected for each treatment (one tree per replicate, 24 trees in total). The experiment included eight treatments: 1) control: no organic fertilizer; 2) cow manure (10 kg/tree in both years); 3) sheep manure (10 kg/tree in both years); 4) chicken manure (10 kg/tree in both years); 5) turkey manure (10 kg/tree in both years); 6) partridge manure (10 kg/tree in both years); 7) quail manure (10 kg/tree in both years); 8) vermicompost (10 kg/tree in both years). Analysis of organic fertilizers was performed before application (Table 3). Fertilizers, in treatment-dependent ratios, were buried in a ditch (20 cm width, 35 cm depth) under the tree canopy at a distance of 1.8 m from the trunk of each tree. Moreover, applications time of fertilizers was mid-November, at the same time as the first rain. The annual rainfall during these years was 374 mm and 395 mm, respectively. The orchard did not receive additional irrigation during the experimental period, relying solely on annual rainfall as the water source.

**Table 3:** Analysis of organic fertilizers used in this experiment (means values ± SD (n = 12) of the two-year data).

Organic fertilizer	pH	Organic matter (%)	N (%)	P (%)	K (%)	Na (%)
Turkey	5.9±1.51	79.8±9.21	4.65±0.98	1.36±0.51	2.78±0.21	0.50±0.1
Quail	8.3±1.12	58.9±5.77	5.87±1.23	1.78±0.19	2.55±0.45	1.46±0.25
Chicken	7.5±0.81	73±8.82	6.50±1.25	1.99±0.93	2.20±0.65	0.4±0.02
Sheep	8±1.83	75±5.72	3.62±0.48	0.68±0.18	3.62±0.87	0.21±0.09
Vermicompost	7.2±1.24	73.5±11.32	1.22±0.35	0.9±0.21	1.10±0.14	0.3±0.11
Partridge	7.8±1.75	71±10.31	4.5±0.74	1.50±0.24	1.50±0.24	0.87±0.15
Cow	7.5±1.24	81.5±2.81	2.26±0.80	0.62±0.12	2.26±0.20	0.15±0.04

### 2.3. Growth measurements

Morphological traits were evaluated after shoot growth stopped (in September) on three scaffolds of trees. Leaf number and syconium number per shoot were calculated by the total number of leaves in the current year’s shoot length. Leaf width (cm) and the current year’s shoot length (cm) were measured using a ruler, while the diameter of new shoots (mm) and inflorescences were measured using a Vernier Caliper (ACCUD 124, Suzhou, China).

### 2.4. Leaf sampling and nutrient determination

To analyze the nutrient status of plants, fifty fully expanded leaf samples of fig from the sixth node from the base of shoots were collected from each of the eight treatments on June 15 (In this time, the caprification (pollination) process and vegetative growth for rain-fed fig trees in Estahban is complete, and fruit set is finishing). Therefore, leaf sampling at this time suitable for determine nutrients status). Fresh leaves underwent a cleaning process with mild detergent, followed by rinsing with distilled water and drying at 70°C until a constant weight was achieved. Subsequently, the dried leaves were ground to pass through a 40-mesh screen. One gram of the ground, dried leaf material was ashed at 550°C for 5 hours. The resulting ash was dissolved in 5 ml of 20% HCl and adjusted to 50 ml with distilled water. Analysis for phosphorus (P) was conducted using a spectrophotometer [3], while potassium (K) was assessed via flame photometry [11]. For nitrogen (N) determination, approximately 0.2 g of dried leaf tissue from each treatment was combusted, and total N was measured using the Kjeldahl method [4].

### 2.5. Fruit quality

#### 2.5.1. Fruit yield and physical properties

Harvesting was conducted from the middle of August until the end of September in both years. The number of fruits per tree and fruit yield for each treatment were determined after harvest. A unique trait of the Sabz cultivar fig is the natural drying of ripe fruits on the tree. Also, the main crop of this variety ripens and undergoes drying directly on the tree by mid-August, with the moisture content of the dried fruit reaching approximately 15%. In general, the appearance quality of dried figs in Iran is classified into three groups based on the skin color of the fruit and whether the fruit is open or closed: 1. Good quality: Fruit with a light skin color and an open ostiole; 2. Medium quality: Fruit with a light brown color and a semi-open ostiole; 3. Poor quality: Fruit with dark skin color and a closed ostiole. Initially, the harvested dried fruit was sorted based on the ostiole opening in figs into three groups: fruit with an open ostiole, semi-open ostiole, and closed ostiole. Additionally, the fruit skin color was assessed using a colorimeter (Chroma Meter CR-400, Minolta, Japan). The color parameters represent whiteness or brightness/darkness (L*), redness/greenness (a*), and yellowness/blueness (b*). Also, fruit was sorted based visually categorized into three color groups: light yellow (good grade), light brown (medium grade), and dark brown (poor grade).

#### 2.5.2. Biochemical properties

Polyphenol extraction followed the modified method established by Bremner [4]. 5 g of powdered fig fruits were macerated in methanol, and after 24 hours in darkness, the extract was filtered and concentrated to dryness using a rotary evaporator. For HPLC analysis, 0.3 g of the dried methanolic extract were mixed with 1000 μl of methanol, filtered through a 0.22 μm pore size membrane, and then injected into the HPLC system (Agilent 1200 model) equipped with a Zorbax Eclipse XDB-C18 column (4.6 × 5 μm i.d.; × 150 mm film thickness, RP) and a photodiode array detector (PDA). Gradient elution, with varying proportions of solvent A (1% formic acid in deionized water) to solvent B (Methanol (v/v)), was employed: Methanol: formic acid 1% (10:90) at 0 min, Methanol: formic acid 1% (25:75) at 10 min, Methanol: formic acid 1% (60:40) at 20 min, and Methanol: formic acid 1% (70:30) at 30 min. Spectra were recorded in the 280 and 320 nm range. The column temperature was maintained at 30°C, and the injection volume was 20 μL, administered automatically via an autosampler. Moreover, the total soluble solids (TSS) value was determined using a refractometer (CETI-Belgium) at 20°C.

### 2.6. Statistical analysis

All data were presented as an average of two years. An analysis of variance was done for the combined analysis of variance across the tests in the two years. Also, data were subjected to analysis of variance (ANOVA) using SAS 9.1. Duncan’s multiple range test was employed to compare means and determine the significance (p > 0.05) among the treatments. The heatmap were generated in ClustVis [5] using the pheatmap packages in R software with default settings.

## Results

### 3.1. The effect of organic fertilizers on vegetative and reproductive growth

According to the results in Table 4, significant differences were observed in shoot growth, shoot diameter, number of leaves, leaf width, and number of syconium among different sources of organic fertilizers. Additionally, Table 4 revealed that shoot growth and diameter primarily increased during late seasons with the use of turkey manure. Treatments involving quail, chicken, sheep, cow, partridge, and vermicompost ranked second in terms of annual shoot growth increase, but their effect on shoot diameter was nearly the same. Moreover, the application of turkey manure led to a significant increase in the number of leaves (7.83) and syconium (6.58) compared to other organic fertilizers. However, the increase in the number of syconium and leaves with other organic manures was minimal and not significantly different from the control treatment. The control treatment recorded the lowest values for shoot length and diameter in the fig trees.

**Table 4:** Effect of different source of organic fertilizers on growth characteristics of rain fed fig tree (mean of the two-year data).

Organic fertilizer	Shoot growth (cm)	Number of leaves	Number of syconium	Leaf width (cm)	Shoot diameter (mm)
Turkey	10.25 a	7.83 a	6.58 a	12.25 a	6.93 a
Quail	8.41 b	5.57 b	4.5 b	11.25 ab	6.17 bc
Chicken	6.66 bc	5.05 b	4 b	9.91 bc	6.46 ab
Sheep	7 bc	5.16 b	4.08 b	9.91 bc	6.16 bc
Vermicompost	7.91 bc	5.41 b	3.75 b	10.41 bc	6.23 bc
Partridge	7.91 bc	5.75 b	3.75 b	10.16 bc	5.92 bc
Cow	7.08 bc	5.66 b	3.66 b	10.25 bc	5.99 bc
Control	6.29 c	5.70 b	3.41 b	9.75 c	5.73 c
*P value*	0.006***	0.003***	0.0002***	0.024*	0.023*

Similar letters in each column indicate nonsignificant differences among organic fertilizer at P > 0.05. For each column, ns, *, **, ***: nonsignificant or significant at P = 0.05, 0.01 or 0.001, respectively.

### 3.2. The effect of organic fertilizers on leaf mineral content of fig trees

Table 5 presents the effect of different sources of animal and bird manure on leaf mineral concentration (K, P, N, and Na). Organic fertilizer treatments significantly influenced the leaf nutrient levels of P, N, and Na in fig leaves, except for leaf K concentration. The P content in fig trees treated with vermicompost (0.124%) was significantly higher than those treated with other organic fertilizers such as partridge (0.115%), cow (0.110%), sheep (0.107%), quail (0.105%), chicken (0.102%), and turkey (0.087%). The highest leaf N value was observed in fig trees treated with chicken (0.88%) and partridge (0.87%), while no significant differences were observed between the control treatment (0.79%) and other organic manures. Moreover, fig trees treated with sheep, partridge, control, turkey, and cow manure had the lowest leaf Na concentration, while the application of some organic manures like quail, chicken, and vermicompost led to an increase in Na concentration in fig leaves.

**Table 5:** Effect of different source of organic fertilizers on mineral concentration in Sabz cultivar fig leaves (mean of the two-year data).

Organic fertilizer	Na (mg L ^-1^)	K (g kg^-1^ DW)	P (%)	N (%)
Turkey	4 b	34.6 a	0.087 b	0.79 ab
Quail	10.6 a	33.6 a	0.105 ab	0.77 ab
Chicken	9 a	36.6 a	0.102 ab	0.88 a
Sheep	3.33 b	30 a	0.107 ab	0.69 b
Vermicompost	6.33 ab	35 a	0.124 a	0.84 ab
Partridge	3.33 b	36.3 a	0.115 ab	0.87 a
Cow	4 b	31.6 a	0.110 ab	0.79 ab
Control	3.66 ab	33.3 a	0.044 c	0.79 ab
*P value*	0.001***	0.85 ^ns^	0.002***	0.043*

Similar letters in each column indicate nonsignificant differences among organic fertilizer at P > 0.05. For each column, ns, *, **, ***: nonsignificant or significant at P = 0.05, 0.01 or 0.001, respectively.

### 3.3. The effect of organic fertilizers on fruit yield attributes

**Fig 1:**
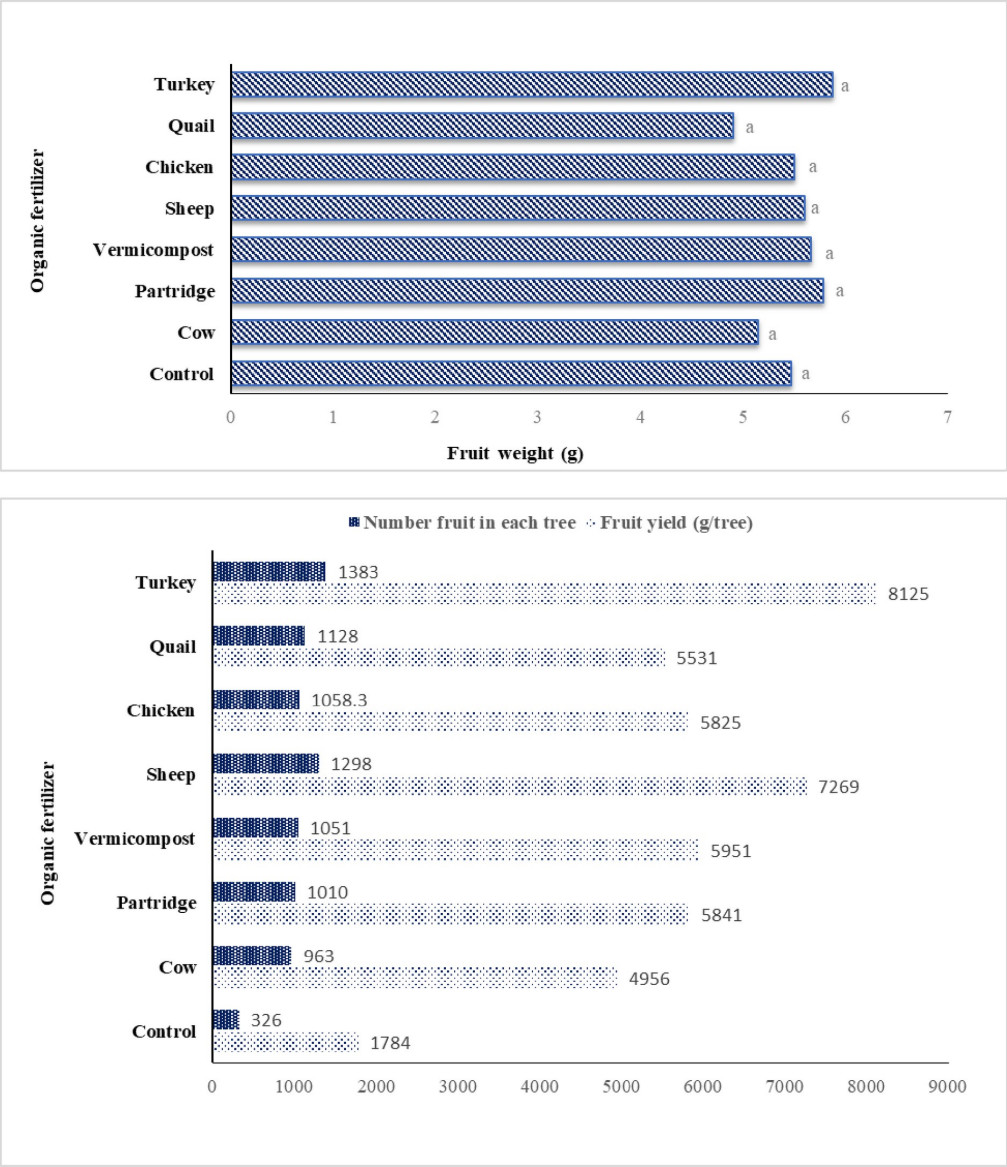
Effect of different source of organic fertilizer on fruit yield, fruit number and fruit weight (mean of the two-year data).

Fig 1 shows the significant effect of organic fertilizers on yield and the number of fruits. Feeding the ’Sabz’ cultivar with all organic manure treatments improved the fruit number and weight compared to the control fig trees. Rain-fed fig trees that received turkey (8125 g), sheep (769 g), vermicompost (5951 g), partridge (5841 g), chicken (5825 g), quail (5531 g), and cow (4956 g) manure had the highest fruit yield compared to the control trees in both study seasons. The number of fruits per tree also significantly increased (P>0.001) in fig trees treated with turkey (1383) and sheep manure (1298), showing a 4-fold increase compared to the control fig tree (326) (Fig 1). However, the data for average fruit weight (Fig 1) showed that trees treated with organic fertilizer had small and no-significant effects.

### 3.4. The effect of organic fertilizers on the physical properties of fig fruits

Table 6 indicates variations in fruit color (L*, a*, and b*) among different organic fertilizers. The L* value was significantly affected by different organic fertilizers in fig dried fruit. The highest L* index was observed in fig trees treated with turkey and cow manure, followed by trees treated with sheep, quail, vermicompost, and partridge. All organic fertilizers, except partridge manure, significantly increased the fruit a* value compared to the control treatment over the two years. Turkey and cow manure were more effective than the other treatments. The lowest L* and a* values were observed in the control treatment. However, organic fertilizers did not significantly affect b* values.

**Table 6:** Effect of different source of organic fertilizer on fruit skin color of rain fed fig orchard based on color meter dates (mean of the two-year data).

Organic fertilizer	L* (brightness/darkness)	a* (redness/greenness)	b* (yellowness/blueness)
Turkey	38 a	4.03 a	15.04 a
Quail	35.34 ab	3.92 a	14.01 a
Chicken	30.09 ab	3.47 a	11.08 a
Sheep	36.52 ab	3.95 a	13.014 a
Vermicompost	34.79 ab	3.41 a	13.34 a
Partridge	31.48 ab	2.98 a	12.14 a
Cow	38.51 a	3.99 a	12.76 a
Control	21.69 b	2.76 a	13.34 a
*P value*	0.02*	0.35^ns^	0.65 ^ns^

Similar letters in each column indicate nonsignificant differences among organic fertilizer at P > 0.05. For each column, ns, *, **, ***: nonsignificant or significant at P = 0.05, 0.01 or 0.001, respectively.

### 3.5. The effect of organic fertilizers on biochemical characteristics of fig fruits

The effect of vermicompost, animal, and bird manures on the biochemical characteristics of fig fruits are shown in Table 7. Some polyphenol compounds, including Trans frolic and coumarin contents of fig fruits, were not influenced by organic fertilizers in the soil at the end of the two years. However, the application of partridge, turkey, and quail significantly increased the Vanillin concentrations compared to the control and other organic fertilizers. The highest (4.12 mg/100 g DW) and lowest hesperidin (1.51 mg/100 g DW) concentrations were observed in fig trees treated with sheep and vermicompost manure, respectively. The use of other organic fertilizers did not have a statistically significant effect on hesperidin content. Moreover, although all sources of organic fertilizers increased the TSS values of dried figs, there were no significant differences between different sources of organic fertilizers in TSS values.

**Table 7:** Effect of different source of organic fertilizers on phytochemical properties of dried fig fruits (mean of the two-year data).

Organic fertilizer	TSS	Gallic acid (mg/100 g DW)	Coumarin (mg/100 g DW)	Vanillin (mg/100 g DW)	Trans frolic acid (mg/100 g DW)	Hesperidin (mg/100 g DW)
Turkey	9.53 a	5.22 ab	0.66 b	2.24 ab	8.01 a	2.64 ab
Quail	9 a	5.11 ab	0.80 b	0.80 ab	8.43 a	3.55 ab
Chicken	9.46 a	2.82 c	0.68 b	1.49 ab	8.43 a	2.43 ab
Sheep	9.53 a	-	0.61 b	2.33 a	8.11 a	4.12 a
Vermicompost	9.40 a	-	0.71 b	0.71 b	8.23 a	1.51 b
Partridge	9.26 a	6.11 a	0.62 b	1.38 ab	8.29 a	2.53 ab
Cow	9.43 a	3.26 c	1.23 a	1.52 ab	9.13 a	2.71 ab
Control	7.90 b	3.93 bc	0.62 b	1.47 ab	7.97 a	2.74 ab
*P value*	0.27 ^ns^	0.003***	0.042*	0.032*	0.47 ^ns^	0.021*

Similar letters in each column indicate nonsignificant differences among organic fertilizer at P > 0.05. For each column, ns, *, **, ***: nonsignificant or significant at P = 0.05, 0.01 or 0.001, respectively.

### 3.6. The effect of organic fertilizers on fig fruit grade

**Fig 2:**
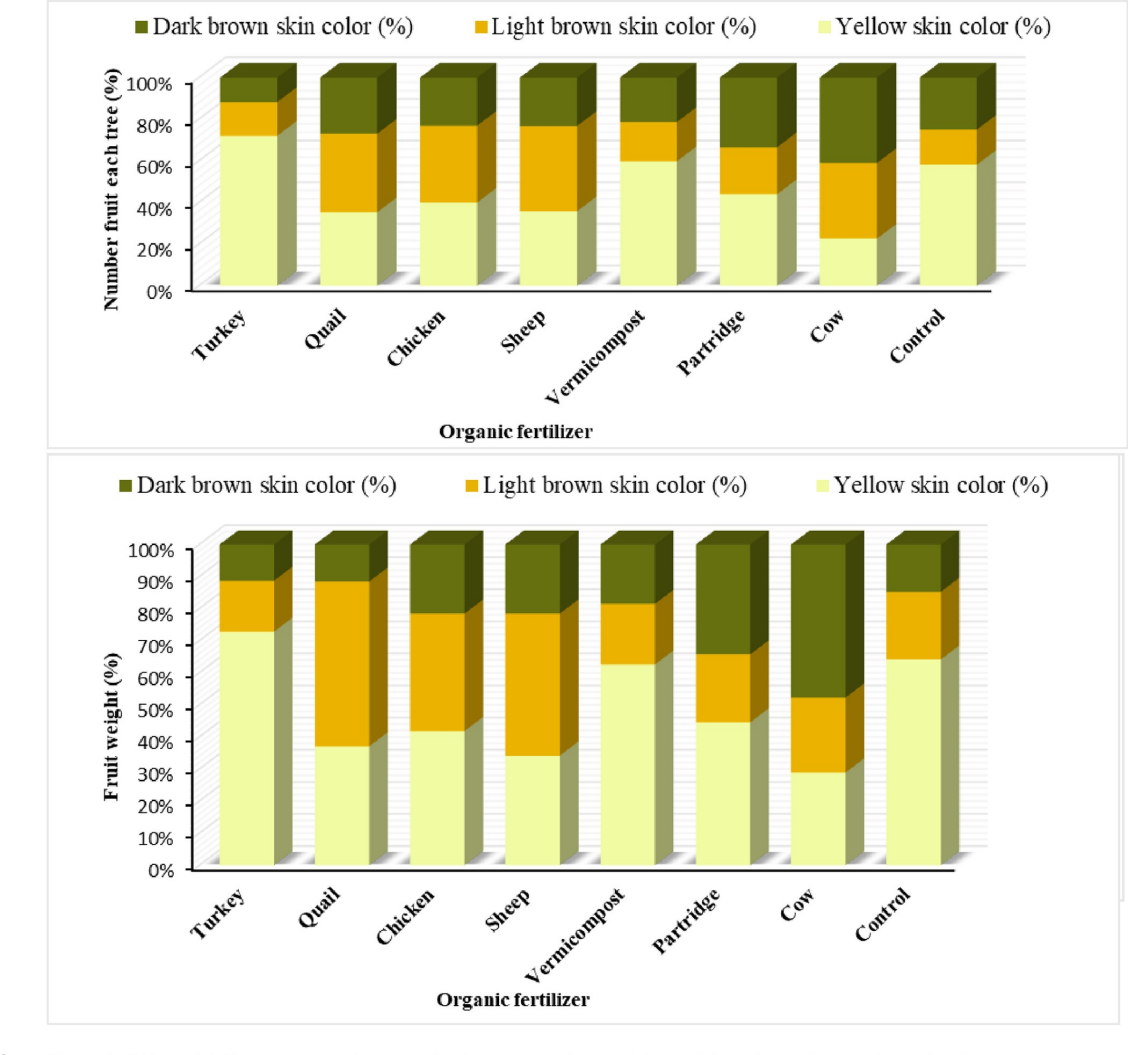
Effect of different source of organic fertilizer on number, weight, and fruit skin color based on visually color (mean of the two-year data).

The application of different sources of organic fertilizers significantly affected the dried fig grade based on visual color in fruit production (Fig 2). The highest percentage of fruits with the best quality (fruit with light yellow skin color) was related to the use of turkey manure (72.07%), while the lowest percentage of best-grade fig fruit was observed in the cow treatment. However, no significant differences were observed between other organic fertilizers treatments and control trees. The effect on the number of fruit weights in each tree with medium quality also showed that the highest fruit weight was 2.5 times greater in fig trees treated with quail (51.35%) compared to control fig trees (19.22%) (Fig 2). The application of sheep (40.95%), quail (37.75%), chicken (36.995%), and cow (36.35%) manure increased the number of fruits with medium quality, whereas the control treatment and other organic fertilizers had lower values (16.88%). For fig fruits with dark brown skin color, except for cow manure, the application of other organic fertilizers decreased the number and weight of the worst quality fruits (fruit with light yellow skin color) in dried figs. The use of turkey manure, on the other hand, decreased the number and weight of the worst quality fruits compared to control and other organic fertilizer treatments (Fig 2).

**Fig 3:**
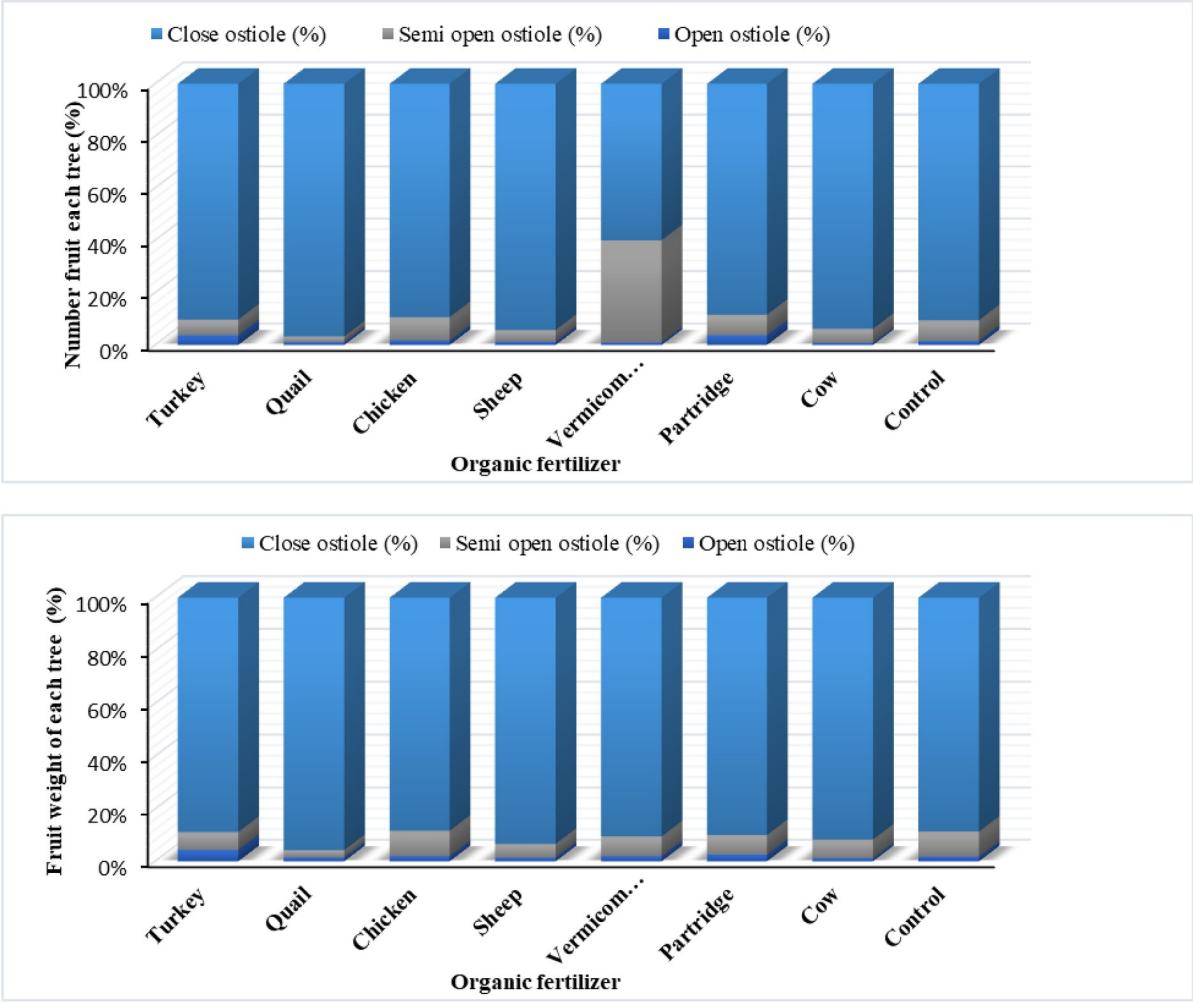
Effect of different source of organic fertilizer on dried fig fruit cracking (mean of the two-year data).

The results indicated that fruit weight and the number of fruits with open, semi, and close ostiole and fruit grade were significantly affected by organic fertilizers (Fig 3). Turkey manure increased the number of fruits with open ostiole in dried figs, while no significant differences were observed in the control and other organic fertilizer treatments in the Sabz cultivar. The highest number (8.90%) and weight (9.58%) of fruits with semi-ostiole grade were observed in fig trees treated with chicken, while the lowest number and weight were found in those treated with quail (2.2%). Partridge manure treatment decreased the number of fruits with close ostiole grade, while the highest fruit weight with close ostiole grade was observed in fig trees treated with quail (95.81%), sheep (93.1%), cow (91.70%), vermicompost (90.60%), and partridge manure (90.60%), respectively (Fig 3). The lowest fruit weight with close ostiole grade was found in the control (89.31%), turkey (88.9%), and chicken (88.43%) treatments.

### 3.7. Correlation analysis

**Fig 4:**
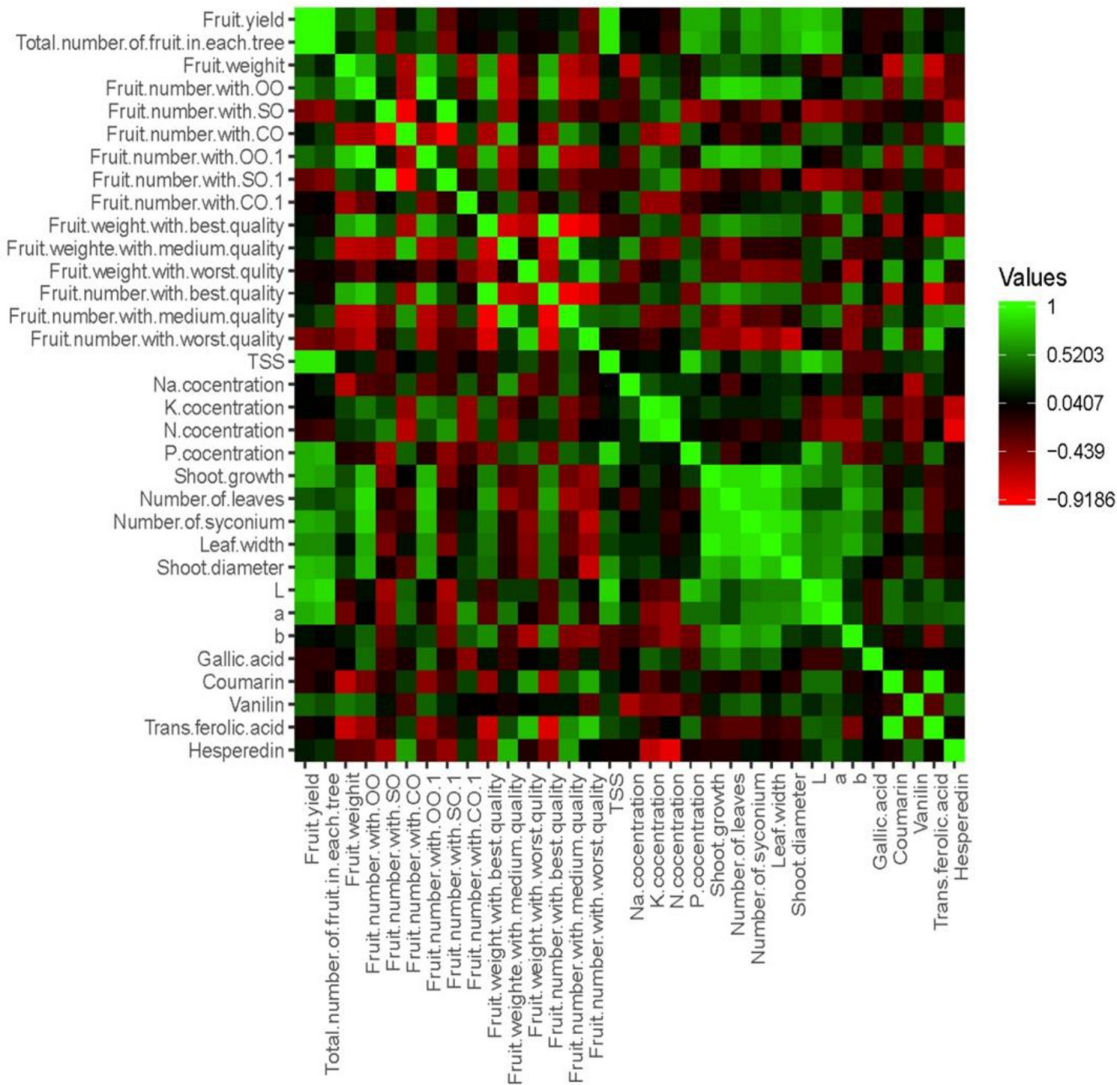
The correlation heatmap of the studied traits in dried fig fruits.

The correlation analysis revealed significant relationships between physic-biochemical fruit characteristics and growth and reproductive traits (Fig 4). Fruit yield and the number of fruits per tree showed the highest significant positive correlation with some growth traits, including the number of syconium, number of leaves, leaf width, annual shoot growth, and shoot diameter. Furthermore, P concentration in leaves exhibited the highest positive correlation with TSS and fruit yield in fig trees. Other physical factors of fig fruits, such as b* skin color, fruit weight, the number of fruits with open ostiole, and fruit weight of open ostiole, had the highest positive relationship with fruit weight of the best quality, while a negative correlation was observed between P concentration in leaves and the number of fruits with close ostiole and fruit weight of the best quality. The main fruit color indexes (L*, a*, and b*) showed the highest positive correlation with growth traits, including the number of syconium, leaf width, annual shoot growth, and shoot diameter, but had a significant negative correlation with N and K concentration in leaves. All polyphenol compounds, except Gallic acid in fruits, exhibited positive correlations with a* fruit color, whereas Gallic acid showed a positive and significant correlation with K concentration.

### 3.8. Cluster analysis

**Fig 5:**
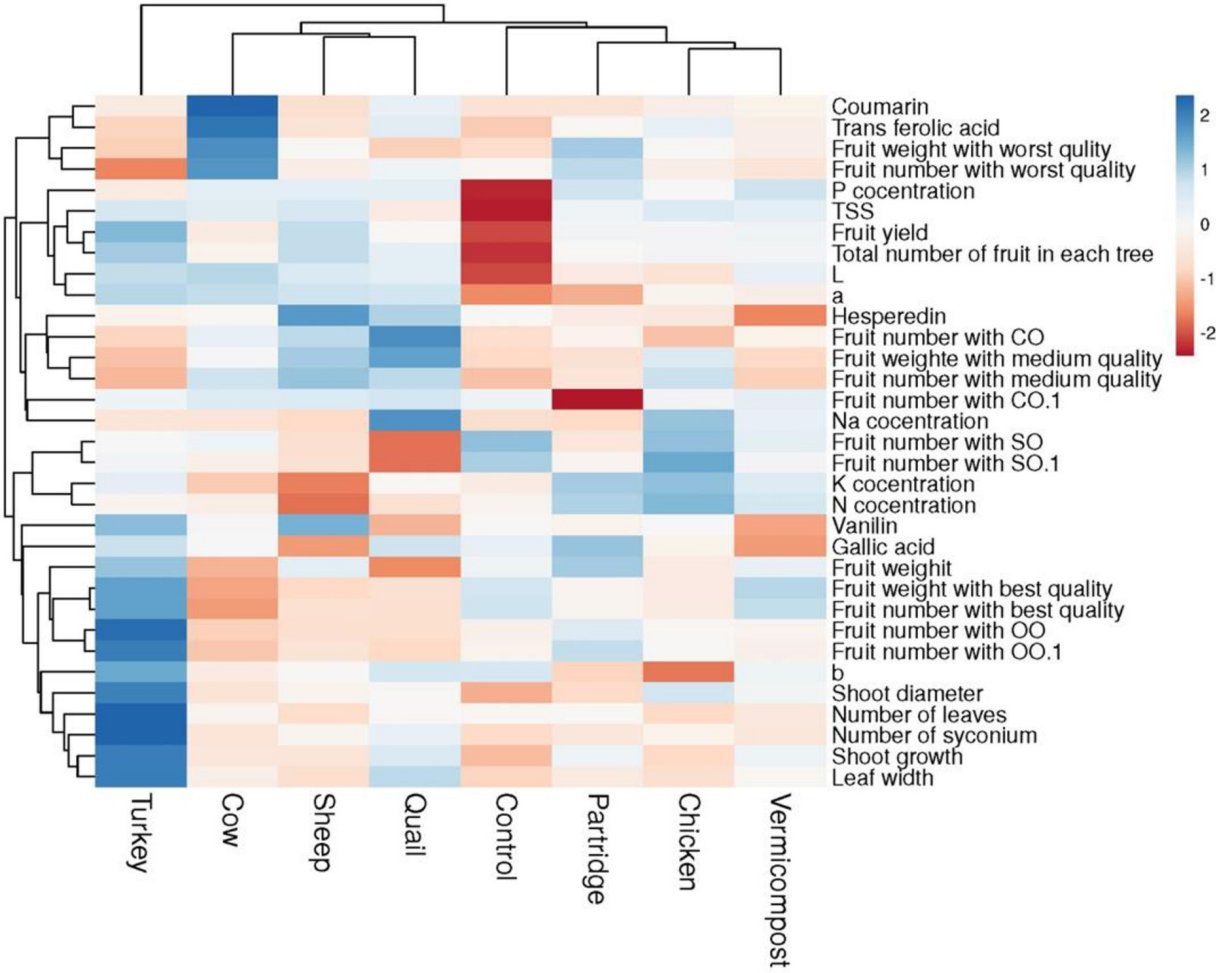
The cluster analyses of different source of organic fertilizers on studied traits in dried fig fruits.

The results of the cluster analysis (Fig 5) categorized various organic manure treatments into three groups based on the investigated tree and fruit characteristics. Turkey manure treatment was positioned in the first group, followed by cow, sheep, and quail manure treatments in the second group. Vermicompost, chicken, partridge manure, and control treatment were grouped in the third cluster. The findings of this cluster analysis provide insights into which organic fertilizers have the most significant influence on fig fruit quality. Turkey manure-treated fig trees showed superiority in most vegetative traits, fruit characteristics such as b* skin color, the number of fruits with open ostiole, fruit number with the best quality, and fruit weight of the best quality. Cow manure application improved some biochemical traits, such as coumarin and transfolic acid. The control treatment and control fig trees showed the lowest values for the main fruit and fig tree characteristics, including P concentration, fruit number per tree, fruit yield, TSS, hesperidins content, L* and b* skin color.

## Discussion

An important parameter that impacts the number of fruits per shoot and consequently affects productivity in fig trees is tree vigor, which is measured as shoot growth, the number of nodes or leaves per shoot, and finally the number of syconium. The improved vigor of the evaluated different organic matter is demonstrated by the longer shoots and increased number of leaves per shoot. Additionally, the results demonstrated that applications of organic fertilizer increased reproductive potential or the number of syconium with rising vegetative growth in fig tree cv. Sabz.

According to botanical knowledge, fig fruit is a vegetative tissue that develops from buds located at each leaf axial of the current season’s growth, and as a consequence, the number of fruits per shoot rises as the number of nodes does. Previous studies have discovered a correlation between such reproductive traits as the plant’s typical ability to produce one fruit per branch node who demonstrated that the application of manure improves the potential production per tree in fig [12]. Adding organic manures can improve the availability and uptake of nutrients from the soil, facilitating development, and having a dramatic impact on the compounds generated by organic manures that regulate plant growth as well as reproductive traits. The significant effect of the compounds released by organic manures that regulate plant growth or the improvement in the availability and uptake of nutrients from the soil that promotes growth may be essential for the improvement of plant growth and reproductive traits brought about by their application. According to the findings, the application of organic fertilizers, particularly those from turkey and quail manure, significantly improves vegetative and reproductive characteristics. However, we found that using other organic matter such as sheep, chicken, and vermicompost had a similar effect on the growth parameters of fig trees. Furthermore, differences in reproductive and vegetative growth resulting from the applied different types of organic matter can also be due to available nutrition derived from the organic fertilizer. These findings conflict with those of Abdel-Nasser and Harhash [6], who discovered that sheep manure was more effective than chicken manure at improving the growth of olive trees. As a result, turkey and quail manure are advised for fig cultivation in arid and semi-arid regions with scarce water resources.

The maximum yield, best quality, and earlier production in fig trees are determined by the soil’s optimum percentage of the three macro-elements, including nitrogen, phosphorus, and potassium. Our findings indicated that except for potassium, all applied organic fertilizers had an impact on macro-elements such as phosphor and nitrogen (Table 4). Mordoğan, Hakerlerler [7] reported that cow and sheep manure did not have important effects on the potassium content of the fig leaf compared to the control trees. They also suggest the utilization of the nutrients added through organic manure for raised vegetative growth and fruit size, thus imposing a dilution effect on the nutrient concentration in leaves. Moreover, analysis of leaf samples showed that the highest level of phosphor concentration was observed in the fig leaves treated with vermicompost, while the lowest value was related to the control fig trees (Table 4). Vermicompost is generally defined as biologically mature peat-like matter, and it is one of the biological organic fertilizers rich in nutrients in plant-available forms such as nitrates, phosphates, exchangeable calcium, and soluble potassium [8]. These results conflict with those who reported the minimum phosphor concentration observed in Vermicompost-treated pomegranate trees. Moreover, another study on fig trees showed that the effect of organic fertilizers, including sheep and cattle manure, on the phosphor concentration of the leaves was not marked. Small but significant differences were observed among different types of organic fertilizers for leaf nitrogen concentration. Fayed [10] found that using a different type of organic manures on ‘Anna’ apple trees increased leaf macro elements (NPK) concentration as compared with control trees.

Our findings provided confidence in the fact that using chicken manure on fig trees caused the maximum N content in fig leaves. These findings are comparable to those of [13], who discovered that fig trees treated with chicken manure had the maximum nitrogen content. Additionally, these findings support those revealed by M. Morsi (2009), who demonstrated that organic manure improved N in date palms. This achievement may be explained by the fact that organic nitrogen sources, like chicken manures, are more challenging to manage than other organic fertilizers because it is very difficult to predict when their nitrogen will become available in the soil, especially in soils with low moisture content [14]. Dry fig yield per tree and fruit number per tree are highly complex categories dependent on genetics, biotic and abiotic factors, and applied orchard management. Fruit yield and fruit number per tree of fig trees significantly increased with all organic fertilizers’ applications in this study (Fig 1). This result may be attributed to the improvement of soil characteristics, mainly macro and micronutrient concentrations, and organic matter of additional organic fertilizers. Moreover, the highest fruit yield in our study, both yields per tree and fruit number per tree, was produced with the turkey manure treatment (Fig 1).

Tofanelli, de Jesus [1] reported that the highest yield values and fruit number per tree were observed in fig trees that were treated by cattle manure fertilization in comparison with control trees. However, we obtained higher fruit yield than we expected compared to cattle and sheep manure yield capacity in fig. The highest fruit yield of the fig trees fertilizers with turkey manure in our study may be due to the increase the number of syconium as the main parameter to determine fruit yield in fig trees. These findings are comparable to those obtained by Leonel and Tecchio [12], that the reported the fertilization of fig trees with organic fertilizers such as cattle manure increased vegetative growth and led to a raised yield of fruits in fig trees. According to AL-Kahtani and Ahmed [15], organic fertilizer increased the rate of fruit sets and decreased fruit falling waves by sustaining enough nutrient content in leaves throughout the trees’ growth cycles to provide the highest yield. Moreover, our results showed that organic fertilizers have an insignificant effect on fruit weight (Fig 1). These findings are similar to the results of Mordoğan, Hakerlerler [7] who observed that the use of some organic manures (sheep and cow) were not affected on fig fruit weight.

The skin color of dry fig fruits relies on the pollen source, droplet formation, air humidity, air temperature and sunlight, soil type, nutritional status of the tree, and moisture. Moreover, light yellow skin color is the most suitable for commercial color among fig color groups [16]. Based on fruit color analysis in dry figs, the use of all organic fertilizers in fig trees improves fruit color by the effect on the L* index. However, the fruits of trees receiving any fertilizers had less yellow color than the control. There are no reports about the effect of different types of organic fertilizer on the skin color of fig fruits. These results are in agreement with Marzouk and Kassem [17] in date fruit, Fallahi and Mohan (2000),and Amiri and Fallahi [18] in apple fruit, who reported that the use of organic fertilizers affected fruit quality characteristics, especially fruit color.

The phenolic compounds are one of the biochemical groups of fig fruits which are the most important for biological applications. These improve human health while also performing a wide range of physiological functions in plants [19–21]. The polyphenol compounds in fig fruits and leaves display various quality attributes. Due to their high concentrations of antioxidants such as phenolic compounds, dry figs are well known for their nutritional value. Chlorogenic acid, Ferulic acid, P-coumaric acid, quinic acid, Cinnamic acid, and Vanillic acid are phenolic acids. Fruits health-promoting qualities are attributed to polyphenols, which enhance the liver, adipose, and skeletal muscle cells’ ability to process glucose and lipids. In his meta-analysis of interventional trials, Aguirre, Arias [22] found that polyphenol consumption has an anti-diabetic effect by increasing the insulin action in skeletal liver and skeletal muscle, reducing plasma free-fatty acid concentrations and hepatic gluconeogenesis, and raising glucose absorption. Ferulic acid also enhances hepatic glycogenesis and insulin sensitivity in diabetic type II rats, although it suppresses glycogenesis [23]. Additionally, Vanillic acid dramatically increased the release of insulin induced by glucose [24]. Therefore, the bioavailability of a substance’s metabolites may differ greatly from that of the parent molecule. Therefore, further information from human clinical studies is required to investigate the bioavailability of polyphenols. This study showed that the use of different sources of organic fertilizers has a significant effect on some polyphenol compounds in dried fig fruit. Also, our results showed that the use of partridge, turkey, and quail significantly increased the Vanillin concentrations concerning the control and other organic fertilizers (Table 7). The effects of different sources of organic fertilizers on the phenols in fig fruit are being studied for the first time in this study.

Ibrahim, Jaafar [25] compared organic fertilization with inorganic fertilization in the medicinal plant (*Labisia pumila* Benth) and resulted the total phenolics, flavonoids, ascorbic acid, saponin, and glutathione synthesis being all increased in this plant. In dried figs, one of the important factors that significantly affect fruit flavor is the total sugar level. Our findings also indicate that improved TSS in mature fig tree orchards did not result from natural fertilizer with organic manure and vermicompost. These results support earlier research that showed the total soluble solids content (%) of fruits from the different doses of manure supplied to the fig trees [1]. The fertilizer combinations employed, ‘Spring Time’ and ‘Red Haven’ did not change considerably in contrast to the control in peach [26]. According to Spinelli, Fiori [27], the quality of the fruit will increasingly be a concern for consumers as they want safe, wholesome, high-nutrition agricultural goods with little to no negative environmental effects. This will open up new market prospects and guarantee better producer earnings. Additionally, customers growing demand for organic fruits leads to an increased need for cutting-edge natural fertilizers, which tends to increase the requirement for different organic fertilizer manufacturing. Our results showed that the highest and lowest percentage of the best quality fruit related to turkey manure (72.07%) and cow treatment, respectively (Fig 2). In addition, no significant effects were observed between other organic fertilizers and the control tree. Wassel, Abdelaal [13] reported that supplying fig trees with some organic manure (sheep and cow) fertilizers enriched with N and biofertilization was responsible for improving yield and fruit quality. The literature reports conflicting findings about the impact of organic fertilization on fruit quality [28]. There is evidence that organic fertilization improves fruit quality in several ways, including fruit color, weight, size, and antioxidant capacity in dates treated with cattle dung and chicken manure [17]. Moreover, According to Mordoğan, Hakerlerler [7] improve by the application of cow and sheep manure mixes.

Based on these findings, it is expected that new organic fertilizers, which are often enhanced with beneficial soil microorganisms, have a helpful effect on the production of good fruit quality in fig orchards. We suggest that to produce fruits of high yield and quality, it needs to be understood how biochemical reactions in plants influence fruit quality. Furthermore, our results showed the highest positive correlation between P concentration in leaves with TSS and Fruit yield in fig trees (Fig 4). Stylianidis and Syrgiannidis [29] reported that fruit soluble solids content (%) of peach trees did not show a significant correlation with any nutrient element. However, in apricots, Bussi, Besset [30] reported that increased K fertilization enhanced fruit soluble solids and coloring. We observed a significant (P > 0.05) increase in fruit yield and the number of fruits. These changes were correlated by increasing the number of the syconium, the number of leaves, leaf width, annual shoot growth, and shoot diameter in fig trees that treated with the organic fertilizers.

We used cluster analysis to show how different organic applications had an effect on the vegetative and reproductive characteristics as well as the patterns of fruit production, both in terms of quantity and quality. The results indicated that the addition of organic fertilizers, especially turkey manure, led to increased vegetative productivity and shifts in the fruit quality of the fig orchard. In general, our results further suggest that the amendment of soil with organic fertilizers provides an effective management practice for improving fruit quality in mature orchards.

### 4.1. In conclusion

The application of organic fertilizers, particularly turkey and quail manure, significantly improves vegetative and reproductive characteristics in fig trees. The results also highlight the impact of different organic fertilizers on the phosphorus, nitrogen, and potassium content in fig leaves, as well as their influence on fruit yield, fruit color, and phenolic compounds in dried figs. The study emphasizes the importance of selecting appropriate organic fertilizers for fig cultivation to achieve higher fruit yield and quality. Further research is needed to better understand the biochemical reactions that influence fruit quality and flavor. Additionally, the study suggests that the utilization of organic fertilizers enriched with beneficial soil microorganisms can enhance fruit quality in mature fig orchards, meeting consumer demands for safe, nutritious agricultural products with minimal negative environmental impacts.

## Supporting information

S1 Data (XLSX)
